# ChatGPT-5.4 in health education: inter-generation stability and persistent readability challenges

**DOI:** 10.3389/fpubh.2026.1864894

**Published:** 2026-07-01

**Authors:** Xingmin Han, Dan Li, Ruirui Xing, Xiaomei Song

**Affiliations:** 1 Institute for Health and Sport, Victoria University, Melbourne, VIC, Australia; 2 The Follow-up Office, The Second Affiliated Hospital of Soochow University, Suzhou, China; 3 Department of Nursing, The Second Affiliated Hospital of Soochow University, Suzhou, China

**Keywords:** ChatGPT-5.4, DISCERN, health information, large language model, patient education, readability, spinal surgery

## Abstract

**Objectives:**

Generative artificial intelligence (generative AI) has been investigated for creating patient education materials (PEMs) to reduce the burden of clinical education and improve health information accessibility. However, prior studies have highlighted limitations in readability, content stability, source transparency, and generation quality. The release of ChatGPT-5.4 provides an opportunity to evaluate the inter-generation stability and usability of a newer frontier model. This study evaluates ChatGPT-5.4's performance in producing PEMs for spinal surgery.

**Method:**

On 5 March 2026, ChatGPT-5.4 was used to address common patient questions about three prevalent spinal surgeries: lumbar disc herniation surgery, spinal fusion surgery, and spinal decompression surgery. Each question generated five independent responses. Qualitative analysis evaluated language sophistication, information depth, structural clarity, and supplementary content. Readability was measured using the Flesch–Kincaid Reading Ease (FKRE), Flesch–Kincaid Grade Level (FKGL), and Simple Measure of Gobbledygook (SMOG). Quality was assessed by two independent reviewers using the DISCERN tool, with the Intraclass Correlation Coefficient (ICC) calculated.

**Results:**

Core medical information remained consistent across generated versions for all three question types, although variations occurred in tone, organization, and detail presentation. The average FKRE ranged from 43.02 to 57.16 (“difficult” to “standard English”), FKGL from 9.12 to 13.34, and SMOG from 8.90 to 11.84, corresponding to reading levels from advanced junior high to early university. The average DISCERN score ranged from 43.2 to 44.9 (“average” quality). ChatGPT-5.4 showed moderate-to-high inter-generation stability with limited content drift in this exemplar context. However because no earlier model was evaluated head-to-head under identical prompts, raters, and time points, this finding should be interpreted as within- study evidence of stability rather than evidence of improved stability over earlier models. Readability remained challenging, and verifiable references were absent.

**Conclusion:**

Within this exemplar spinal-surgery context, ChatGPT-5.4 demonstrated moderate-to-high inter-generation consistency in lexical content and core medical themes. Complex language and lack of traceable references may limit accessibility and patient trust.

**Practice implications:**

ChatGPT-5.4 may support spinal-surgery patient education by generating stable, clinically plausible PEMs, though factual accuracy was not independently verified. Readability remains above recommended health- literacy levels, and clinician review and plain-language optimization are required before patient use.

## Introduction

A substantial body of research has evaluated the utility of generative AI for producing patient education materials (PEMs), motivated primarily by the potential to reduce the time burden of clinical counseling, lower health literacy barriers, and mitigate inequities in information access (1–6). Typical applications include preoperative informed consent, postoperative rehabilitation guidance, chronic disease self-management, and medication adherence education (1–4). However, these studies have consistently identified several common limitations of AI-generated PEMs, including elevated readability levels, phrasing that may lead to misunderstanding, instability across repeated generations, factual inaccuracies or outdated knowledge, and the absence of verifiable references. These limitations are particularly important in clinical education contexts, where patients may rely on written information to understand treatment options, risks, recovery expectations, and postoperative self-management.

On 5 March 2026, OpenAI released GPT-5.4 in ChatGPT as GPT-5.4 Thinking, as well as in the API and Codex (7). GPT-5.4 was introduced as a frontier model designed for professional work, with improvements in reasoning, coding, agentic workflows, document-heavy tasks, tool use, and factual reliability (7). According to OpenAI, GPT-5.4 produces more consistent and polished results for real-world professional knowledge work, including tasks involving spreadsheets, presentations, and documents (7). The release materials also report reductions in false claims and response-level factual errors compared with GPT-5.2 (7). Although these improvements were not specifically validated for patient education in the release materials, they are highly relevant to the generation of PEMs, which requires stable explanations, coherent structure, accurate medical content, and language that can be understood by non-specialist readers.

Against this background, ChatGPT-5.4 may offer advantages for producing health education materials, particularly through improved consistency, factual reliability, and the ability to handle document-style outputs. However, improved general model capability does not automatically guarantee suitability for patient-facing clinical communication. PEMs require not only accurate content, but also appropriate readability, balanced discussion of benefits and risks, transparent sourcing, and alignment with patients' health literacy levels. Therefore, before ChatGPT-5.4 can be considered a meaningful improvement for clinical education, its performance should be systematically evaluated in specific medical contexts.

Building on prior work evaluating the quality of AI-generated PEMs for spinal surgeries (1), the present study examines the performance of ChatGPT-5.4 in producing health education materials within an exemplar spinal-surgery context. We adopted the core framework of previous evaluations while strengthening methodological rigor, with a particular focus on assessing readability, content quality, and inter-generation stability. We hypothesized that ChatGPT-5.4 would (i) demonstrate high inter-generation stability in core medical content, while (ii) readability would remain above recommended patient-education standards, and (iii) content quality would remain limited by incomplete source attribution and variable coverage of treatment alternatives.

## Methods

This study employed ChatGPT-5.4 (OpenAI) to generate patient education materials. Spinal surgery was selected as the clinical context for testing. Lumbar disc herniation surgery, spinal fusion surgery, and spinal decompression surgery were chosen as common spinal procedures spanning a range of invasiveness, from decompression to fusion. They were also the procedures used in a prior comparative evaluation of AI-generated spinal-surgery PEMs (1), allowing comparison with that study's findings.

On 5 March 2026, ChatGPT-5.4 was used to address frequently asked patient questions concerning three commonly performed spinal surgical procedures (Table 1). These prompts were designed to elicit accessible, user-friendly medical information from the AI system, following the approach described by previous study (1, 8). No additional prompt engineering (e.g., system instructions, role settings, or formatting constraints) was applied, as this approach was intended to reflect real-world clinical use where both patients and clinicians typically do not have formal training in prompt construction. To evaluate the stability of ChatGPT-5.4 in generating patient education materials across repeated prompts, we conducted a multi-round generation test for each of the three spinal surgery questions (Q1–Q3). Each question was entered into ChatGPT-5.4 independently five times under identical conditions, with no additional clarifying prompts or contextual information provided between attempts. To prevent context carryover or personalization effects from influencing inter-generation comparisons, each of the five generations per question was run in a new, separate chat session with memory and personalization disabled. No information from earlier prompts, responses, or attempts at the same question was therefore available to the model during generation.

**Table 1:** Questions for spinal surgery terminology clarification.

Prompt
*Q1: I need information about “*Lumbar Disc Herniation Surgery”* as I am unfamiliar with medical terms. Can you help clarify that term for me?*
*Q2: I need information about “*Spinal Fusion Surgery”* as I am unfamiliar with medical terms. Can you help clarify that term for me?*
*Q3: I need information about “*Spinal Decompression Surgery”* as I am unfamiliar with medical terms. Can you help clarify that term for me?*

## Measures

### Stability analysis

The DISCERN instrument, developed by D. Charnock (9), is a validated tool for assessing the quality of written health information, with initial validation in myocardial infarction, endometriosis, and chronic fatigue syndrome. It is widely used across medical fields, including evaluations of spinal surgery information (1, 10, 11), and is among the most frequently applied instruments for this purpose.

DISCERN contains three sections: (1) eight items assessing information reliability, (2) seven items evaluating the completeness and accuracy of treatment details, and (3) one item providing an overall quality rating. Each item is scored from 1 (“very poor”) to 5 (“very high”), giving a total score of up to 80. Scores above 70 indicate “excellent” quality, while those above 50 are considered “fair.” Previous studies have used DISCERN to assess AI-generated health education materials (e.g., (12, 13)). In this study, it was applied to evaluate the quality of AI-generated responses.

To quantify response stability across repeated generations, lexical overlap was assessed using Jaccard similarity for all pairwise comparisons among the five responses generated for each question (14). Because five responses were produced for each question, this yielded 10 unique response pairs per question. Before calculating similarity, all responses were standardized through basic text preprocessing: all text was converted to lowercase, punctuation marks were removed, and common stop words with limited semantic value (e.g., articles, auxiliary verbs, and conjunctions) were excluded. The remaining content words in each response were then represented as a set of unique words, such that repeated occurrences of the same word within a response were counted only once. For each response pair, Jaccard similarity was calculated as the number of shared unique words divided by the total number of unique words appearing across both responses. This metric ranges from 0 to 1, where higher values indicate greater lexical overlap between the two responses. Pairwise Jaccard scores were first calculated separately for each of the 10 response pairs within a question and were then averaged to produce a mean Jaccard similarity score for that question. This mean score was used as a quantitative indicator of inter-generation stability, with higher values interpreted as reflecting greater consistency in lexical content across repeated generations. To aid interpretation, Jaccard similarity scores were described as follows: values below 20% indicated low overlap, 20–40% low-to-moderate overlap, 40–60% moderate overlap, 60–80% high overlap, and values above 80% very high overlap, approaching near-verbatim wording consistency (14).

### Readability

The readability of the responses was assessed using three well-established metrics: the Flesch–Kincaid Reading Ease (FKRE), the Flesch-Kincaid Grade Level (FKGL), and the Simple Measure of Gobbledygook (SMOG) (15, 16). The FKRE assigns a numerical score ranging from 0 to 100, with higher values indicating simpler and more accessible text. A score near 100 suggests that the content is very easy to understand, whereas lower scores denote increased complexity. The FKGL is an adaptation of the FKRE that estimates the minimum education level required for comprehension, expressed in U.S. school grade levels (15). A higher FKGL score corresponds to more complex text, implying that individuals with lower levels of formal education may find the material difficult to understand (15). The SMOG index also estimates the years of education needed for comprehension, but it focuses on the frequency of polysyllabic words (three or more syllables) within a standard 30-sentence sample (16). Higher SMOG scores indicate greater complexity and are particularly sensitive to technical or specialized vocabulary. All readability metrics were calculated using the WebFX online readability test (17), which applies established readability formulas. To enhance reproducibility, all FKRE, FKGL, and SMOG scores were then independently recalculated and validated manually using their original published formulas. In general, patient education materials are recommended to be written at approximately the 6–8th grade reading level to ensure accessibility for the general population (18).

### Statistical analysis

Qualitative analysis was performed by summarizing and comparing the presence of predefined key content elements across the five generated responses for each question (Q1–Q3) in four dimensions: language Level, Information Depth, Structural Clarity, and Additional Content. Two raters (XH and RX) independently evaluated all responses, with disagreements resolved by a third researcher (XS). Descriptive statistics were calculated for readability metrics (FRES, FKGL, SMOG), sentence count, word count, and proportion of complex words. DISCERN scores were summarized as means from two independent reviewers (XH and RX), and inter-rater reliability was assessed using the intraclass correlation coefficient (ICC) with a two-way random-effects model. Specifically, because the analysis used the mean DISCERN score across the two reviewers, a two-way random-effects, average-measures, absolute-agreement ICC [ICC(2, k)] was calculated, together with its 95% confidence interval. All analyses and visualizations were performed in R language.

## Results

### Qualitative analysis

The qualitative analysis of five generated responses for the three questions (Q1–Q3) were shown in Tables S1–3. All the responses can be found in Additional file 1. Across the results, the core medical information remained highly consistent, covering terminology definitions, pathophysiology or fundamental surgical principles, common indications, major surgical techniques, surgical objectives, and recovery periods, reflecting ChatGPT-5.4's stability in conveying essential medical content. However, variations in the presentation of details were observed across different generations. In terms of language level, responses for all three procedures ranged from plain, analogy-based explanations (e.g., describing intervertebral disks as “soft, cushion-like pads” or likening spinal fusion to “welding”) to extensive use of technical anatomical or clinical terminology (e.g., “nucleus pulposus,” “arthrodesis,” “foramina”), with analogies most often employed to explain anatomical structures or underlying causes. With regard to information depth, the level of detail varied substantially between versions: some responses provided only general statements (e.g., “surgery may be considered when symptoms are severe or conservative treatment is ineffective”), whereas more detailed versions included time thresholds (e.g., “after 6–12 weeks of unsuccessful conservative therapy”), urgent indications (e.g., cauda equina syndrome, bladder or bowel dysfunction), rare surgical techniques (e.g., endoscopic discectomy, corpectomy), or potential risks (e.g., fusion failure, cerebrospinal fluid leakage). In terms of structural clarity, responses included both well-organized, numbered or segmented formats and more loosely structured continuous prose, with the “dual-layer structure” (beginning with a plain-language explanation followed by technical details) considered by reviewers to be the clearest format. In terms of additional content, responses for all three questions occasionally included suggestions for visual aids, pros-and-cons comparisons, or explanations of differences between surgical types.

Jaccard similarity results are presented in Table S4. Mean pairwise Jaccard similarity ranged from 54.10 to 63.48%, indicating moderate to high lexical stability across repeated generations. Consistent with the qualitative analysis, ChatGPT-5.4 responses retained a broadly similar core vocabulary when the same question was repeatedly entered, further supporting the relative stability of lexical content across attempts.

### Readability

**Figure 1:**
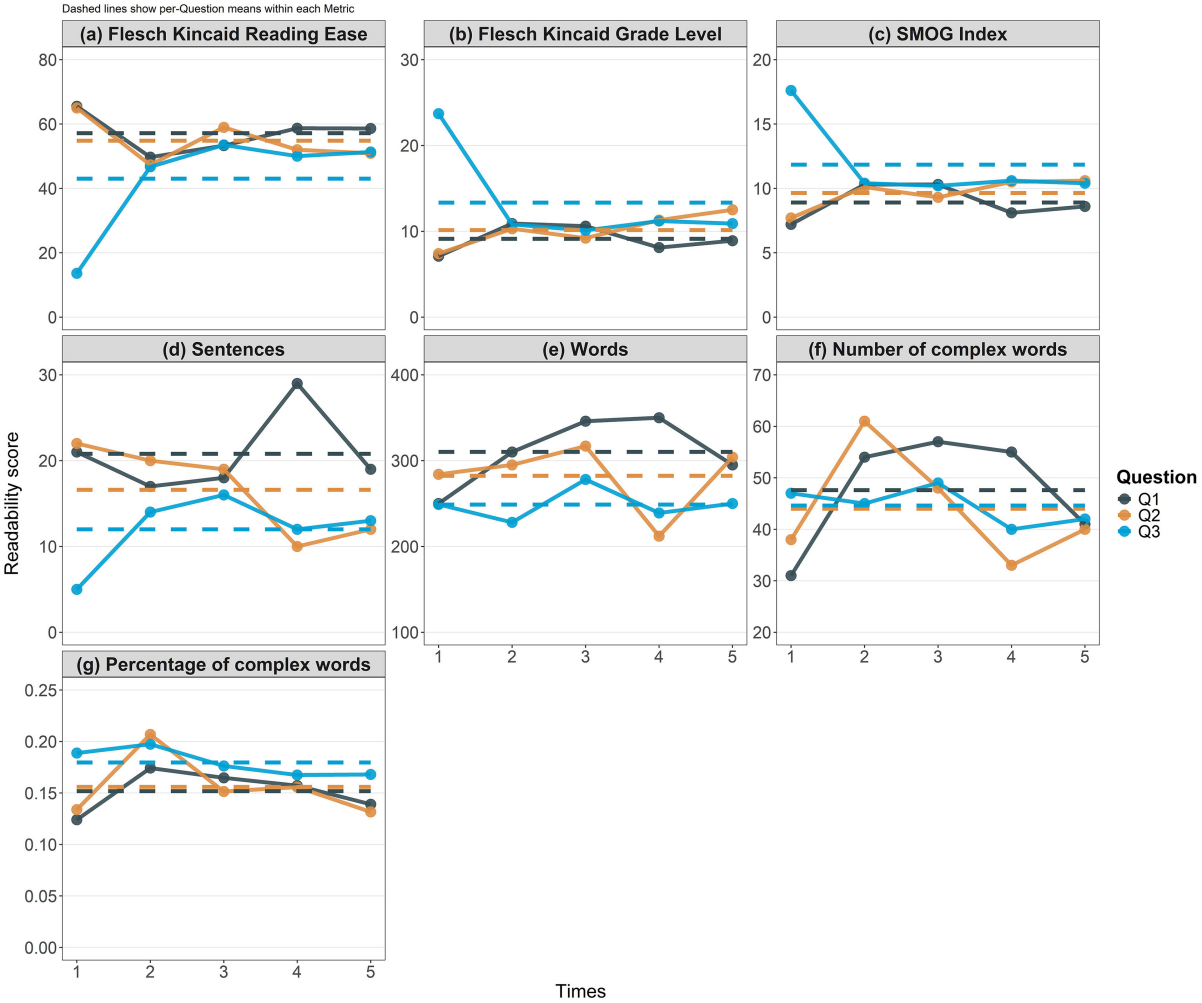
Readability results for each question across five attempts.

Readability outcomes are presented in Figure 1, where the dashed line denotes the mean readability score across the five ChatGPT-generated responses (Table S5, additional file 2). Subplots a, b, and c display the distributions of the FKRE, FKGL, and SMOG Index scores, respectively. Overall, the average readability scores were comparable across the three questions. The mean FKRE values ranged from 43.02 to 57.16, indicating readability levels from “fairly difficult” to “plain English.” The mean FKGL values ranged from 9.12 to 13.34, corresponding to comprehension levels from lower secondary school (Grade 9) to early university (Grade 13). SMOG Index scores (mean range: 8.90–11.84) were consistent with the FKGL results. Across all three questions, the first generated response was the most difficult to read (e.g., for Q3, FKRE = 13.6), whereas responses from the second to fourth attempts exhibited greater readability (FKRE range: 46.7–53.5) and remained relatively stable thereafter.

Subplots d and e illustrate the number of sentences and words per generated response (Table S5, additional file 2). Sentence counts ranged from 5 to 29, while word counts ranged from 212 to 350. The number of complex words varied more substantially, ranging from 31 to 61 per response; however, the proportion of complex words was comparatively stable, ranging from 12.40 to 20.68%, with mean values consistently between 15 and 20% (Figure 1, subplot f and g).

### DISCERN score

**Figure 2:**
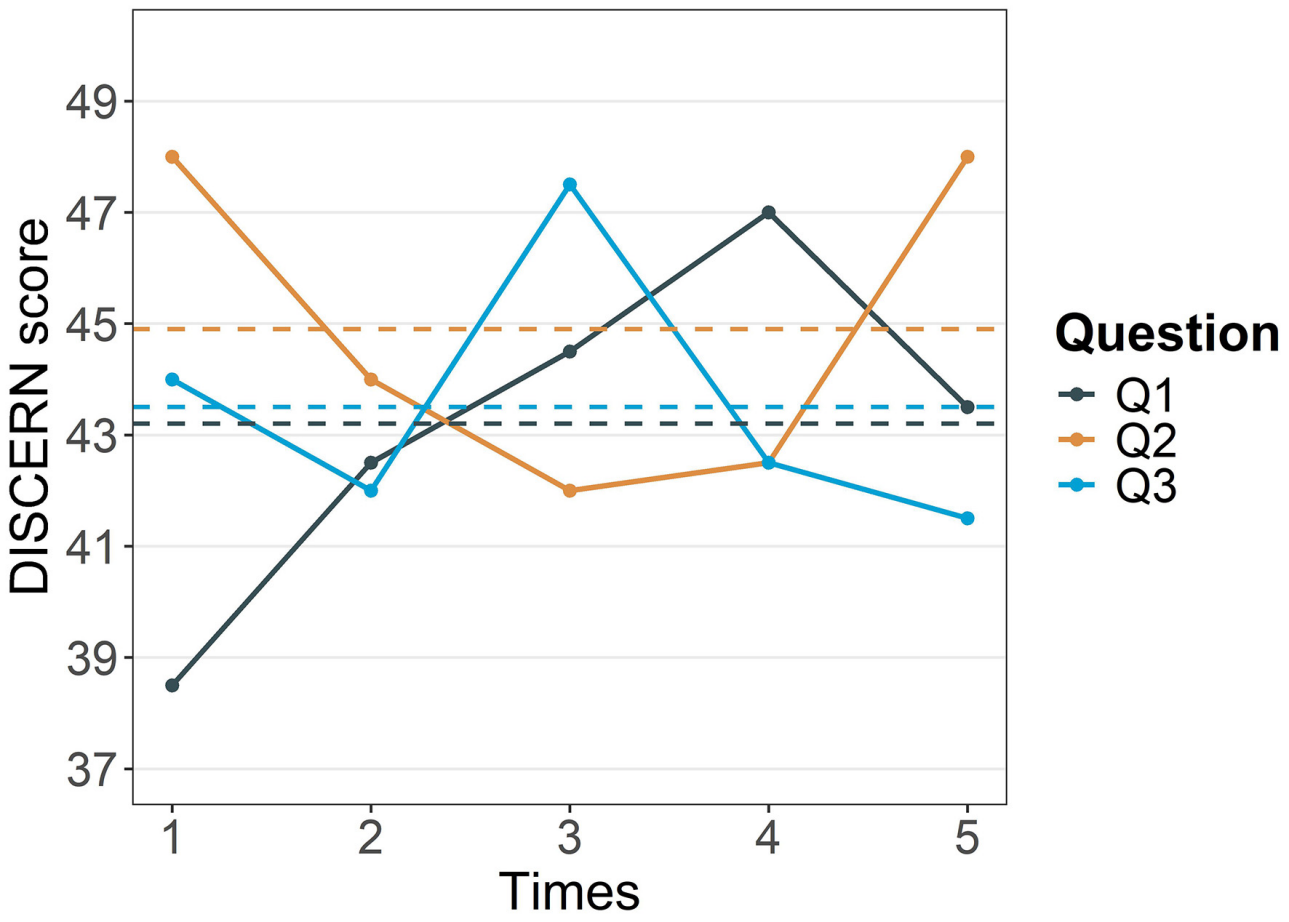
DISCERN scores for each question across five attempts.

Two independent reviewers (XH and RX) evaluated each generated response using the DISCERN instrument. The mean scores from both reviewers were calculated and are presented in Figure 2 (Table S6, additional file 2). The inter-rater reliability, as measured by the intraclass correlation coefficient [ICC(2, k); two-way random-effects, average-measures, absolute agreement], was 0.81 [95% CI (authors to insert from R output)], indicating good absolute, indicating agreement between the two reviewers. The DISCERN scores were highly consistent across the three questions, with mean values ranging from 43.2 to 44.9 and an overall score range of 38.5 to 48. These results indicate that the quality of ChatGPT-5.4's outputs for patient education materials was relatively uniform across different surgical topics and generation attempts. However, the mean scores fall within the “fair” quality range according to DISCERN classification, suggesting that while the content was generally relevant and clinically plausible (though its factual accuracy was not independently verified, it may lack the completeness, depth, or referencing expected for high-quality health information in clinical contexts.

Further examination of the DISCERN results suggests that the moderate scores were primarily driven by several recurring limitations. First, the absence of verifiable references or explicit source attribution consistently reduced scores on items related to information reliability, as the generated responses did not allow readers to independently verify the accuracy or currency of the content. Second, although risks and complications were sometimes mentioned, their presentation was inconsistent across responses, with some versions providing only brief or partial discussion of potential adverse outcomes. Third, the coverage of treatment alternatives was often incomplete, with limited systematic comparison between different surgical options or consideration of non-surgical management strategies.

## Discussion

### Overview of our findings

This study evaluated ChatGPT-5.4's performance in generating PEMs for three common spinal-surgery topics: lumbar disc herniation surgery, spinal fusion surgery, and spinal decompression surgery. Across repeated generations, ChatGPT-5.4 consistently provided core medical information, including terminology definitions, pathophysiology or basic surgical principles, common indications, major surgical techniques, surgical goals, and general recovery expectations. The Jaccard similarity results and qualitative assessment both suggest that the model maintained moderate to high inter-generation stability in lexical content and medical themes.

However, the findings also show that improved stability does not necessarily translate into optimal patient-facing communication. Although the core content remained broadly consistent, individual responses varied in tone, structural organization, level of technical detail, and inclusion of supplementary explanations. Readability remained above recommended health-literacy levels, with FKGL and SMOG scores indicating that many responses required at least secondary-school to early university reading ability. DISCERN scores were relatively stable but remained in the moderate range, largely because the responses lacked verifiable references, inconsistently discussed risks and complications, and did not systematically compare surgical and non-surgical alternatives.

Overall, these findings partly support the prespecified hypotheses. ChatGPT-5.4 showed promising stability in repeated generations, but persistent limitations in readability and source transparency remain important barriers to direct clinical use.

### Comparison with previous study

Previous studies have evaluated earlier ChatGPT models and other LLMs for patient education in spine-related and broader medical contexts. These studies generally found that newer models could generate coherent and relevant health information, but that readability, completeness, hallucination risk, and lack of references remained recurring limitations (1, 19–24). For example, prior work comparing ChatGPT 3.5 and 4.0 in low back pain education reported improvements in response quality and consistency, while also identifying persistent challenges in readability and psychosocial contextualization (19). That study found ChatGPT 4.0 scored significantly higher than 3.5 on response quality and DISCERN-based reliability, though readability for both versions stayed above recommended patient-education levels (19). Our results follow a similar pattern: ChatGPT-5.4's DISCERN scores fell in the “fair” range (43.2–44.9 of 80) with high inter-generation stability, while readability (FKGL Grade 9–13) again exceeded recommended levels. Across these model generations, content reliability and consistency appear to be improving incrementally, while readability for general patient audiences remains largely unresolved. Similar concerns have been reported in studies of AI-generated PEMs for colorectal cancer, prostate cancer, dermatology, and neurosurgical conditions (20–24, 33–35).

The present findings are broadly consistent with this literature. ChatGPT-5.4 appears to provide more stable repeated outputs than patterns reported for some earlier models, particularly in maintaining a consistent set of core medical concepts across multiple attempts. No earlier model, however, was tested head-to-head under the same prompts, raters, and time point as this study. The apparent improvement therefore reflects an indirect comparison with a literature that differs in prompt wording, evaluator composition, and assessment period, and should be read as broadly consistent with—rather than proof of—an improvement over earlier models. One possible explanation is the broader gains in professional knowledge-work generation and factual consistency reported for GPT-5.4 (7), though without a direct comparison this remains speculative. Nevertheless, the readability results show that model advancement alone does not fully resolve the health-literacy problem. In this study, ChatGPT-5.4 generated responses that were often concise and information-dense, but this concision may have increased conceptual load by concentrating medical terminology into shorter passages.

The findings also suggest that stability and accessibility should be treated as separate dimensions of performance. A model may provide consistent information while still producing text that is too complex for many patients. Similarly, a response may be medically relevant but still receive only moderate DISCERN scores if it does not clearly identify sources, explain uncertainty, compare treatment options, or discuss risks in a balanced way. Therefore, evaluations of LLM-generated PEMs should go beyond factual correctness and include readability, source transparency, treatment balance, and user comprehension.

### Interpretation of ChatGPT-5.4's performance

ChatGPT-5.4's relatively stable delivery of core spinal-surgery information may reflect improvements in generating structured professional documents and maintaining consistency across repeated tasks. Several responses used a “plain-language first, technical explanation later” structure, which is potentially useful for patient education. For example, the model sometimes explained intervertebral disks as “cushion-like pads” or described spinal fusion using familiar analogies such as “welding.” These strategies can reduce initial cognitive burden and may help patients approach unfamiliar medical terminology.

At the same time, the model frequently shifted from accessible explanation to dense anatomical or procedural terminology, such as “nucleus pulposus,” “foramina,” “arthrodesis,” or “corpectomy.” Although these terms are clinically appropriate, they may limit comprehension when not carefully defined or when introduced too quickly. This pattern helps explain why readability scores remained higher than recommended levels despite the model's apparent attempt to communicate in patient-friendly language.

Another important finding is the absence of verifiable references. In clinical education, source transparency is central to patient trust and safety. Without references to clinical guidelines, hospital resources, or peer-reviewed evidence, patients and clinicians cannot easily verify the accuracy, currency, or applicability of generated content. This limitation is consistent with prior research on LLM-generated health information and highlights the need for retrieval-augmented generation, citation mechanisms, or clinician review before patient use.

The DISCERN scores here also need to be read alongside the prompts that produced them. DISCERN evaluates how well consumer health information covers treatment options, balances risks and benefits, and discloses its sources. Our prompts, however, asked ChatGPT-5.4 only to clarify an unfamiliar surgical term—not to address any of this. The “fair” scores may therefore say as much about this mismatch between prompt and instrument as about the model's underlying medical knowledge: a prompt that explicitly asked for treatment alternatives, risks, and sources might well have scored higher without any change to the model itself. Future DISCERN-based comparisons should take this into account, either by checking whether the prompt actually solicits the content DISCERN evaluates, or by designing prompts specifically for that purpose.

### Research implications

This study suggests that ChatGPT-5.4 may be useful as a preliminary drafting tool for spinal-surgery PEMs, particularly where clinicians need a quick starting point for explaining common procedures. Its relatively stable generation of core content may reduce the risk of major content drift across repeated use. However, the model should not be treated as a ready-to-use patient education system without further adaptation.

For clinical implementation, several safeguards are needed (25–32). First, prompts should explicitly request plain-language explanations at a defined reading level, such as Grade 6 to Grade 8. Second, prompts should require balanced discussion of treatment alternatives, including non-surgical options where relevant. Third, generated materials should include traceable sources or be linked to validated institutional guidelines. Fourth, clinician review should remain essential before distributing AI-generated materials to patients.

The readability limitations identified here point to prompt engineering as another way of making AI-generated PEMs more accessible. The wording and framing of a prompt can meaningfully affect how readable ChatGPT's medical responses are (8). Asking for an explanation at a specific reading level (for example, Grade 6–8), requesting that technical terms be defined as soon as they appear, or asking for a short plain-language summary followed by optional technical detail, could all help reduce the conceptual density seen in our results. Simplification can, however, go too far: prompts that demand minimal vocabulary or very short answers risk leaving out warning signs, risks, or treatment alternatives. What is needed is systematic testing of iterative or template-based prompts that balance readability against completeness and accuracy, rather than a single blanket instruction to simplify.

From a research perspective, future studies should compare ChatGPT-5.4 with earlier models under identical prompts to quantify whether improvements in stability, readability, and content quality are statistically meaningful. Studies should also assess patient-centered outcomes, such as comprehension, trust, anxiety reduction, decision confidence, and recall of key information. Such outcomes are necessary to determine whether technical improvements in LLM outputs translate into real clinical benefit.

### Practical implications

In real-world clinical settings, ChatGPT-5.4-generated PEMs may be best positioned as clinician-supervised draft materials rather than standalone patient resources. For example, clinicians could use the model to generate an initial explanation of a procedure, then revise the output to match local practice, patient literacy level, and individual clinical circumstances. This approach may reduce the time burden of preparing education materials while preserving clinical oversight.

An FKGL of Grade 9–13 matters for patients with limited health literacy. Because a substantial share of adults read below the high-school level, materials at the upper end of this range may be out of reach for many of the patients they are intended for. Such readers may misunderstand or only partly grasp explanations of surgical indications, risks, and recovery expectations, with knock-on effects for informed consent, adherence to postoperative instructions, and recognition of warning signs that need urgent attention. Because older adults, non-native speakers, and people with less formal education already face greater barriers to healthcare, these readability gaps could widen rather than narrow existing health disparities. AI-generated content should therefore be paired with clinician review, plain-language adaptation, or follow-up verbal counseling before it reaches patients.

The results also indicate that institutions should not rely on generic prompts alone. Instead, standardized prompt templates may be needed to ensure that outputs consistently include the purpose of surgery, who may benefit, alternatives, risks, recovery expectations, warning signs, and questions patients should ask their clinicians. Such templates could help reduce variability in structure and improve the clinical usefulness of generated materials.

### Strengths and limitations

To our knowledge, this study provides one of the first evaluations of ChatGPT-5.4 for generating PEMs in a spinal-surgery context. Its strengths include the use of multiple clinically relevant questions, repeated generations for each prompt, qualitative content assessment, readability metrics, Jaccard similarity analysis, and DISCERN-based quality evaluation. Together, these methods provide a multidimensional assessment of model performance.

Several limitations should be acknowledged. First, this study focused only on spinal surgery, and the findings may not generalize to other specialties, conditions, or patient populations. Second, all prompts and responses were in English, limiting applicability to non-English-speaking contexts. Third, the study assessed textual properties and reviewer-rated quality, but did not directly measure patient comprehension, satisfaction, trust, or decision-making outcomes. Fourth, we did not formally measure hallucination frequency or severity, although this is an important concern for AI-generated health information. Undetected factual errors could matter clinically: an inaccurate description of a surgical risk, recovery timeline, or warning sign might leave a patient with unrealistic expectations, delay them from seeking care for a genuine complication, or lead to a decision based on incorrect information, particularly where such materials are used without clinician oversight. Future evaluations of AI-generated patient education materials should therefore include systematic hallucination checks, ideally with clinicians fact-checking against current guidelines. For the same reason, where this manuscript describes content as “clinically plausible” or “relevant,” this reflects the reviewers' qualitative judgment based on their medical knowledge, not confirmation that every factual statement was checked against current clinical guidelines. Fifth, because large language models change over time, our findings reflect ChatGPT-5.4's performance at one point in time and may not generalize to later versions or system updates. Sixth, Jaccard similarity captures lexical overlap, not semantic equivalence: two responses could use entirely different wording for the same clinical content, yielding a low Jaccard score despite consistent meaning, or could share much of the same vocabulary while differing in emphasis, completeness, or accuracy, yielding a high score despite differing content. Semantic similarity methods, such as sentence-embedding cosine similarity, could complement lexical measures in future work.

### Future research directions

Future research should evaluate ChatGPT-5.4 across a wider range of medical specialties, clinical scenarios, and patient populations. Direct comparisons with earlier models, including ChatGPT-4-series models and other contemporary LLMs, are needed to determine whether GPT-5.4 provides measurable improvements in stability, readability, factual reliability, and clinical usefulness. Cross-linguistic and cross-cultural studies are also needed, as readability and patient comprehension may vary substantially across languages and health systems.

Future studies should also move beyond text-based metrics and include patient-centered outcomes. Experimental studies could test whether ChatGPT-5.4-generated PEMs improve patient understanding, reduce decisional conflict, support informed consent, or influence adherence to postoperative instructions. In addition, research should examine whether prompt engineering, retrieval-augmented generation, and clinician-in-the-loop workflows can reduce readability barriers and improve source transparency.

## Conclusion

This study evaluated ChatGPT-5.4's performance in generating spinal-surgery PEMs. The model consistently delivered core medical information across repeated generations and demonstrated moderate to high inter-generation stability. However, readability remained above recommended patient-education standards, and the absence of verifiable references limited overall quality and trustworthiness. These findings suggest that ChatGPT-5.4 may be useful as a clinician-supervised drafting tool for patient education, but its outputs require further optimization for readability, source transparency, and patient comprehension before direct clinical use.

## Data Availability

The original contributions presented in the study are included in the article/Supplementary material, further inquiries can be directed to the corresponding author/s.
