# Supplementary material for: ChatGPT-5.4 in health education: inter-generation stability and persistent readability challenges
Source: Front Public Health. 2026 Jul 1;14:1864894. doi: 10.3389/fpubh.2026.1864894 (PMC13369432; doi:10.3389/fpubh.2026.1864894)
Supplement: Supplementary file 1 [file Table_1.DOCX]

**Additional file 1 Chatgpt 5 response results**

**Q1, I need information about “Lumbar Disc Herniation Surgery” as I am unfamiliar with medical terms. Can you help clarify that term for me?**

**Attempt 1**

Sure — I can break it down simply and then give you a bit more detail.

**1. Breaking down the term**

- **Lumbar** → Refers to the *lower back* area of the spine.
- **Disc** → Soft, cushion-like pads between the bones (vertebrae) of the spine that act as shock absorbers.
- **Herniation** → When the soft inner part of the disc bulges or leaks out through a tear in its tougher outer layer.
- **Surgery** → A medical operation performed to fix the problem.

**2. What it means**
“Lumbar

disc herniation surgery” is an operation done to treat a slipped or ruptured disc in the lower back. The goal is to remove or repair the part of the disc that is pressing on nearby nerves, which can cause **back pain**, **leg pain (sciatica)**, numbness, tingling, or weakness.

**3. Why it’s done**

- Usually, herniated discs are treated first with rest, physiotherapy, and medications.
- Surgery is considered when symptoms are **severe**, **persistent**, or **causing nerve damage** (e.g., weakness, loss of bladder/bowel control).

**4. Common surgical types**

- **Discectomy** → Removes the part of the disc pressing on the nerve.
- **Microdiscectomy** → Same as above but done through a small incision using a microscope.
- **Laminectomy** → Removes part of the bone over the spinal canal to relieve pressure.

**5. Recovery**

- Most people go home the same day or next day.
- Light activities may start within days, but full recovery can take weeks to months depending on the case.

If you like, I can also prepare **a simple diagram of the lumbar spine and herniated disc** so you can visualize it better.

**Attempt 2**

Sure. I’ll explain **“Lumbar Disc Herniation Surgery”** in simple terms first, then give you a more medical explanation so you can understand it clearly.

**Plain-English Explanation**

- **Lumbar** → the lower part of your spine (your lower back area).
- **Disc** → soft, cushion-like pads between the bones (vertebrae) in your spine that act like shock absorbers.
- **Herniation** → when the inner gel-like material of a disc bulges or leaks out through a tear in the tougher outer layer.
- **Surgery** → a medical operation to fix the problem.

**So**, “Lumbar Disc Herniation Surgery” means an operation done on the lower back to repair or remove part of a spinal disc that has slipped, bulged, or ruptured and is pressing on nerves, often causing pain, numbness, or weakness in the legs.

**More Detailed Medical View**

- **Condition**: A lumbar disc herniation happens when the **nucleus pulposus** (soft inner part) of a spinal disc protrudes through the **annulus fibrosus** (tough outer ring). In the lumbar region, this often compresses the **sciatic nerve roots**, causing **sciatica** (shooting pain down the leg).
- **When surgery is considered**:
  - Severe leg or back pain that doesn’t improve with rest, medication, or physiotherapy.
  - Significant weakness, numbness, or difficulty controlling bladder/bowel function (emergency case: *cauda equina syndrome*).
- **Types of Surgery**:
  - **Discectomy / Microdiscectomy** – removal of the herniated portion of the disc to relieve pressure on the nerve.
  - **Laminectomy** – removal of part of the vertebral bone (lamina) to create space for the nerves.
  - **Spinal fusion** – sometimes done if there’s instability, joining two or more vertebrae together.
- **Goal**: Reduce nerve compression, relieve pain, and restore mobility.

If you want, I can also make you a **simple diagram** showing where the lumbar disc is and what happens during surgery — it can make the term much easier to remember. Would you like me to do that?

**Attempt 3**

Sure — I’ll break it down in simple terms first, then give you a more medical-level explanation.

**Simple explanation**

- **Lumbar** = the lower part of your spine (lower back).
- **Disc herniation** = one of the soft, cushion-like discs between your back bones (vertebrae) has bulged or ruptured, pressing on nearby nerves.
- **Surgery** = an operation done to fix the problem, usually by removing or repairing the part of the disc that is causing pressure.

So, **lumbar disc herniation surgery** is a procedure to relieve pressure on spinal nerves in the lower back caused by a damaged or slipped disc.

**More medical detail**

- **What happens in lumbar disc herniation?**
  The spine is made up of bones (vertebrae) separated by discs that act as shock absorbers. If part of a disc’s soft inner material pushes out through a tear in the tougher outer layer, it’s called a **herniated disc**. In the lumbar area, this can press on the **sciatic nerve** or other spinal nerves, causing lower back pain, leg pain (sciatica), numbness, or weakness.
- **Why surgery is done:**
  Surgery is considered if:
  - Pain or nerve symptoms are severe and don’t improve with rest, medication, or physical therapy.
  - There is weakness, loss of bladder/bowel control (emergency), or nerve damage risk.
- **Common surgical techniques:**
  - **Microdiscectomy** – removing the part of the disc that’s pressing on the nerve through a small incision, often using a microscope.
  - **Laminectomy** – removing part of the vertebral bone (lamina) to give the nerve more space, sometimes combined with disc removal.
  - **Endoscopic discectomy** – using a camera and small instruments through a tiny incision.
- **Goal:**
  Relieve nerve pressure → reduce pain, restore movement, and prevent permanent nerve damage.
- **Recovery:**
  Many people can go home the same or next day, with gradual return to normal activities in a few weeks, though full healing may take months.

If you’d like, I can also **compare the different surgical options for lumbar disc herniation**, including pros, cons, and recovery times, so you can see them side-by-side. Would you like me to do that?

**Attempt 4**

Sure — let’s break it down in plain terms first, then go into more detail.

**1. What is lumbar disc herniation?**

- Your spine is made up of bones called *vertebrae*, and between them are *discs* that act like cushions or shock absorbers.
- These discs have a soft, jelly-like center (the *nucleus pulposus*) surrounded by a tougher outer ring (the *annulus fibrosus*).
- **Lumbar** refers to the lower back region.
- A **lumbar disc herniation** happens when part of that soft center pushes out through a tear in the outer ring.
- This can press on nearby spinal nerves, causing pain, numbness, tingling, or weakness in the lower back and legs (commonly called *sciatica*).

**2. What is lumbar disc herniation surgery?**

- It’s an operation done to **relieve pressure** on the affected spinal nerve(s) caused by the herniated disc.
- The goal is to remove the piece of disc material that’s pressing on the nerve while preserving as much healthy disc as possible.

**3. Common surgical types**

- **Microdiscectomy**:
  - The most common and minimally invasive.
  - Uses a small incision and a microscope or magnifying tools to remove the herniated part of the disc.
- **Laminectomy (or laminotomy)**:
  - Removes part of the bone (lamina) over the spinal canal to create more space and access the disc.
- **Endoscopic discectomy**:
  - Uses an endoscope (tiny camera) through a small incision to remove disc material.

**4. When is surgery considered?**
Usually after:

- 6–12 weeks of non-surgical treatments (rest, physiotherapy, medications) fail to relieve symptoms.
- Severe pain that limits daily activities.
- Muscle weakness or loss of bladder/bowel control (urgent cases).

**5. Recovery**

- Hospital stay can be same-day or 1–2 nights, depending on the approach.
- Walking is encouraged soon after surgery.
- Light activities in days to weeks; full recovery may take 4–12 weeks.
- Success rates are high (about 80–90% get significant relief), but there’s still a chance of re-herniation.

If you want, I can also make you a **simple diagram** showing what a herniated disc looks like and what the surgery removes — that usually makes it much easier to picture. Would that help?

**Attempt 5**

Sure — let’s break down the term **“Lumbar Disc Herniation Surgery”** step by step in plain language, without assuming medical knowledge.

**1. Lumbar**

- Refers to the **lower back** area of your spine.
- The lumbar spine consists of **five vertebrae (L1–L5)** located between your rib cage and your pelvis.
- This region carries much of your body’s weight and is a common site for back problems.

**2. Disc Herniation**

- Between each pair of vertebrae is a **spinal disc** — a soft, cushion-like structure that acts as a shock absorber.
- Each disc has:
  - **A soft, jelly-like center** (nucleus pulposus)
  - **A tougher outer layer** (annulus fibrosus)
- **Herniation** means:
  - The outer layer tears or weakens.
  - Part of the soft inner material pushes out.
- This can press on **nearby spinal nerves**, causing:
  - Back pain
  - Leg pain (sciatica)
  - Numbness or weakness in the legs

**3. Surgery**

- If symptoms don’t improve with rest, physical therapy, or medication, doctors may suggest surgery.
- The **goal**: remove the part of the disc pressing on the nerve and relieve symptoms.
- Common lumbar disc herniation surgeries include:
  - **Microdiscectomy** – a minimally invasive procedure to remove the herniated part.
  - **Laminectomy** – removal of part of the bone (lamina) to make space for the nerve.
  - Sometimes **spinal fusion** is done if the spine needs extra stability.

**4. Putting It Together**

**Lumbar Disc Herniation Surgery** =
An operation on the **lower back** to remove or repair part of a **damaged spinal disc** that is pressing on nerves, with the aim of reducing pain, numbness, or weakness.

If you like, I can also give you a **simple diagram** showing where the lumbar discs are and what herniation looks like — that often makes it much easier to understand. Would you like me to do that?

**Q2 *: I need information about “*Spinal Fusion Surgery*” as I am unfamiliar with medical terms. Can you help clarify that term for me?***

**Attempt 1**

*Of course — I’ll explain it step by step in plain language.*

***1. Breaking down the term***

- ***Spinal*** *→ Related to the spine (your backbone), which is made up of bones called vertebrae.*
- ***Fusion*** *→ Joining or “fusing” things together so they become one solid piece.*
- ***Surgery*** *→ A medical operation.*

***2. What it means*** *“Spinal fusion surgery” is an operation where* ***two or more bones in your spine are permanently joined together****. This stops movement between them, which can reduce pain or correct certain spine problems.*

***3. Why it’s done*** *Spinal fusion may be recommended for:*

- ***Degenerative disc disease*** *(worn-out discs causing pain)*
- ***Spinal instability*** *(bones moving too much)*
- ***Severe scoliosis or kyphosis*** *(curved spine)*
- ***Fractures or injury*** *to the spine*
- ***Tumors or infections*** *that weaken spinal bones*

***4. How it’s done***

- *The surgeon places* ***bone graft*** *(bone tissue from your body or a donor, or synthetic material) between the vertebrae.*
- *Over time, the bone grows together — like how broken bones heal — making one solid bone.*
- *Metal plates, screws, or rods are often used to hold the bones steady while they fuse.*

***5. Effects***

- *The fused part of your spine won’t move anymore, which can reduce pain but also* ***reduces flexibility*** *in that area.*
- *It’s meant to stabilize and protect the spinal cord and nerves.*

***6. Recovery***

- *Hospital stay is usually a few days.*
- *It can take* ***months*** *for the bones to fully fuse.*
- *Physical therapy is often needed to strengthen muscles and improve movement.*

*If you want, I can also give you a* ***comparison table between spinal fusion surgery and lumbar disc herniation surgery*** *so you can clearly see the differences. That might make the terms easier to remember.*

**Attempt 2**

*Sure — I’ll break it down in plain terms first, then give you the more medical explanation.*

***Plain-language explanation***

*Spinal fusion surgery is a medical procedure where two or more bones in your spine (called* ***vertebrae****) are permanently joined together so they can’t move independently.
It’s a bit like “welding” the bones — surgeons use bone or a bone-like material between the vertebrae, and sometimes add metal plates, screws, or rods to hold everything steady while the bones grow together.
The goal is to make that part of the spine solid, which can help reduce pain, correct deformities, or improve stability.*

***Medical definition***

***Spinal fusion*** *is an* ***orthopaedic surgical procedure*** *designed to achieve* ***arthrodesis*** *(fusion) of adjacent vertebral segments. It involves:*

- ***Bone grafting*** *— using autograft (patient’s own bone), allograft (donor bone), or synthetic bone substitutes.*
- ***Internal fixation*** *— instrumentation like rods, screws, plates, or cages to maintain alignment and immobilize the segment.*
- ***Fusion process*** *— over several months, new bone tissue grows across the graft, uniting the vertebrae into one continuous bone.*

***Why it’s done***

*Spinal fusion may be performed to:*

- *Treat* ***spinal instability*** *(e.g., from fractures, severe arthritis, or spondylolisthesis).*
- *Correct* ***spinal deformities*** *(e.g., scoliosis or kyphosis).*
- *Relieve pain from* ***degenerative disc disease*** *or certain types of spinal stenosis.*
- *Stabilize the spine after removing a damaged disc or tumour.*

***Key points to know***

- ***Permanent****: Once fused, the vertebrae no longer move independently.*
- ***Recovery****: Bone fusion takes months; physical therapy is often needed.*
- ***Risks****: Infection, nerve injury, failure to fuse (non-union), or reduced flexibility.*

*If you’d like, I can also make a* ***simple diagram*** *showing what spinal fusion looks like compared to a normal spine — that usually makes it much easier to picture.*

**Attempt 3**

*Sure — I’ll break it down in clear, non-technical language first, then give you a slightly more medical explanation so you understand both sides.*

***In simple terms***

*Spinal fusion surgery is an operation where two or more bones in your spine (called vertebrae) are permanently joined together. The surgeon places bone or a bone-like material between them, and often uses metal screws, rods, or plates to hold everything in place while it heals.*

- ***Goal:*** *Stop movement between those bones.*
- ***Why:*** *To reduce pain, improve stability, or correct a deformity.*

***Why it’s done***

*Doctors may recommend spinal fusion for:*

- ***Spinal instability*** *– when vertebrae move too much or slip out of place (spondylolisthesis).*
- ***Severe degeneration*** *– such as advanced arthritis or degenerative disc disease.*
- ***Deformities*** *– like scoliosis (curved spine) or kyphosis (hunched spine).*
- ***Injuries*** *– fractures or severe damage to the spine.*
- ***Spinal cord compression*** *– from conditions like stenosis (narrowing of the spinal canal).*

***How it works (basic steps)***

1. ***Bone graft****: The surgeon uses a piece of bone (either from your own body or a donor) or an artificial substitute.*
2. ***Placement****: The graft is placed between two vertebrae.*
3. ***Stabilization****: Metal rods, screws, or plates are added to keep the vertebrae from moving while the bone heals.*
4. ***Healing (fusion)****: Over a few months, the bone graft grows together with the vertebrae, forming a single solid bone.*

***What it means for you after surgery***

- *The fused part of the spine no longer bends or twists.*
- *This can relieve pain or prevent further damage, but it can also slightly limit flexibility in that section of the back.*
- *Recovery can take several months, and physical therapy is often needed.*

*If you’d like, I can also give you a* ***diagram and recovery timeline*** *so you can visually see how spinal fusion works and what to expect afterward. Would you like me to prepare that?*

**Attempt 4**

*Sure — I’ll explain it clearly and in plain language.*

***Spinal fusion surgery*** *is an operation done to permanently join (or “fuse”) two or more bones in your spine, called* ***vertebrae****.*

- *In a healthy spine, the vertebrae are separated by discs and joints that allow movement and flexibility.*
- *In spinal fusion, the surgeon places bone or a bone-like material between the vertebrae, and sometimes uses metal plates, screws, or rods to hold them together while the bone heals.*
- *Over time, the bone grows and the vertebrae become one solid piece — so there is* ***no movement between them anymore****.*

***Why it’s done*** *Spinal fusion may be recommended to:*

- *Treat spinal instability (e.g., due to fractures, severe arthritis, or slipped vertebrae)*
- *Correct deformities (like scoliosis)*
- *Relieve pain from certain spinal conditions (such as degenerative disc disease)*
- *Stabilize the spine after removing a damaged disc or tumor*

***Key points***

- *It can reduce pain and prevent further damage, but it also* ***reduces flexibility*** *in the fused part of the spine.*
- *Recovery usually involves weeks to months of healing, and physical therapy is common afterwards.*

*If you’d like, I can also give you a* ***simple diagram*** *showing what spinal fusion looks like compared to a normal spine — that often makes it much clearer.*

**Attempt 5**

*Sure — let’s break it down into simple, clear terms first, then add a bit of medical detail so you get the full picture.*

***Spinal Fusion Surgery — in plain language***

- *It’s an* ***operation on the spine****.*
- *The goal is to* ***join (fuse) two or more bones of your spine*** *(called vertebrae) together so they heal into a single solid piece.*
- *This stops movement between them, which can reduce pain or correct certain spinal problems.*

*Think of it like* ***welding two shaky parts together*** *so they no longer move independently.*

***Why it’s done***

*Doctors may recommend spinal fusion if:*

- *You have* ***spinal instability*** *(the bones move too much)*
- *There is* ***severe degeneration*** *from arthritis or wear-and-tear*
- *Conditions such as:*
  - ***Degenerative disc disease***
  - ***Scoliosis*** *(sideways curvature of the spine)*
  - ***Spondylolisthesis*** *(one vertebra slips over another)*
  - ***Fractures***
  - ***Spinal deformities***
  - ***Chronic back pain*** *from instability*

***How it works***

1. ***Access the spine*** *— via an incision in the back, side, or front of the body.*
2. ***Prepare the bones*** *— remove damaged discs or cartilage between vertebrae.*
3. ***Insert bone graft*** *— small pieces of bone (from you or a donor, or synthetic material) are placed between the vertebrae.*
4. ***Stabilise*** *— metal screws, rods, or plates are often used to hold the bones steady while they fuse.*
5. ***Healing*** *— over several months, the bone graft grows together with the vertebrae, forming one solid bone.*

***Pros & cons in brief***

***Benefits****:*

- *Reduces pain caused by movement in damaged segments*
- *Corrects deformity or instability*

***Risks****:*

- *Infection, bleeding*
- *Nerve damage*
- *Failure of the bones to fuse fully (non-union)*
- *Reduced flexibility in the fused section*

*If you’d like, I can also make a* ***simple diagram*** *showing what happens before and after spinal fusion so it’s even easier to visualise. Would you like me to prepare that?*

***Q3: I need information about “Spinal Decompression Surgery” as I am unfamiliar with medical terms. Can you help clarify that term for me?***

**Attempt 1**

Certainly!
**Spinal decompression surgery** is a general term for surgical procedures that **relieve pressure** on the spinal cord or the nerves coming out of it. This pressure—often called *compression*—can be caused by conditions such as:

- **Herniated or slipped discs** (when a disc between vertebrae bulges or ruptures)
- **Spinal stenosis** (narrowing of the spinal canal)
- **Bone spurs** from arthritis
- **Tumors**
- **Injuries** that cause swelling or misalignment

**What the surgery aims to do**

The goal is to **free up space** in the spinal canal or nerve openings so that nerves are no longer pinched. This can reduce symptoms such as:

- Pain (in the back, neck, arms, or legs)
- Numbness or tingling
- Weakness in muscles
- Difficulty walking or standing

**Common types of spinal decompression surgery**

- **Laminectomy** – removes part of the vertebral bone (lamina) to widen the spinal canal
- **Laminotomy** – removes only a small portion of the lamina
- **Discectomy** – removes part of a damaged disc pressing on nerves
- **Foraminotomy** – enlarges the openings where nerves exit the spine

**Key points**

- It’s usually considered **only after non-surgical treatments** (like physiotherapy, medication, or injections) haven’t worked
- Can be done in the **neck (cervical spine), upper/mid-back (thoracic spine), or lower back (lumbar spine)**
- Recovery time and risks depend on the type of surgery, your overall health, and the underlying problem

If you like, I can also **compare spinal decompression surgery with spinal fusion surgery** so you can see how they differ—they’re often mentioned together. Would you like me to do that?

**Attempt 2**

Sure — let’s break it down in plain language, then touch on the key medical details.

**Spinal decompression surgery** is an operation that aims to **relieve pressure on the spinal cord or nerves**.

- The **spinal cord** is a bundle of nerves running down your back, protected by the spine’s bones (vertebrae).
- Sometimes, conditions like a **herniated disc**, **bone spurs**, **spinal stenosis** (narrowing of the spinal canal), or **tumors** can press on these nerves. This can cause pain, numbness, weakness, or difficulty moving.

**How it works:**

- The surgeon removes or reshapes the part of bone, ligament, or disc that is pressing on the nerve.
- There are different techniques depending on the cause and location:
  - **Laminectomy** – removes part of the vertebral bone (lamina).
  - **Discectomy** – removes part of a herniated disc.
  - **Foraminotomy** – widens the openings where nerves exit the spine.

**Goal:**

- Reduce pain, restore movement, and prevent further nerve damage.

**When it’s considered:**

- Usually after other treatments (medications, physical therapy, injections) haven’t helped enough.
- If there are signs of serious nerve compression, like loss of bladder/bowel control or severe weakness, surgery may be urgent.

If you want, I can also make a **simple diagram** showing what happens during spinal decompression surgery so it’s easier to visualize.

**Attempt 3**

Sure. **Spinal decompression surgery** is a type of operation designed to **relieve pressure on the spinal cord or the nerves branching from it**.

Here’s a clear breakdown:

**1. Why it’s done**

- Your spine has bones (vertebrae) stacked like blocks, with a canal in the middle that houses the spinal cord and nerve roots.
- Sometimes, **parts of the spine press on these nerves**—this can happen due to:
  - **Herniated discs** (disc material bulging out)
  - **Bone spurs** from arthritis
  - **Thickened ligaments**
  - **Spinal injuries**
  - **Tumors** or abnormal growths
- This pressure can cause **pain, numbness, weakness, or tingling**, often in the back, arms, or legs.

**2. What the surgery does**

The goal is to **create more space** for the spinal cord and nerves so that the pressure is reduced.
This can involve:

- **Removing part of the bone** (e.g., laminectomy – removal of the lamina, the back part of a vertebra)
- **Trimming or removing herniated disc material**
- **Shaving down bone spurs**
- Sometimes combining with **spinal fusion** (joining vertebrae to stabilize the spine)

**3. Types of spinal decompression surgery**

- **Laminectomy** – removes the lamina to open up space
- **Discectomy** – removes part of a damaged disc
- **Foraminotomy** – widens the openings where nerves exit the spine
- **Corpectomy** – removes part or all of a vertebral body (less common)

**4. Expected benefits**

- Relieves nerve pain (e.g., sciatica)
- Improves mobility
- Reduces or stops numbness/weakness

**5. Risks**

- Infection
- Bleeding
- Nerve injury
- Spinal fluid leak
- Incomplete relief or recurrence of symptoms

If you’d like, I can also make a **simple diagram** showing where and how spinal decompression surgery works so it’s easier to visualize.
Would you like me to draw that?

**Attempt 4**

Sure — I’ll break it down in plain language first, then give you a bit more medical detail so you can see where it fits in healthcare.

**Plain Explanation**

**Spinal decompression surgery** is an operation doctors do to relieve pressure on the spinal cord or the nerves coming out of it.
This pressure can happen when something inside your spine is squeezing or crowding the nerves — for example:

- A bulging or herniated disc
- Bone growths (spurs) from arthritis
- Thickened ligaments
- A spinal injury or tumor

When nerves are under pressure, it can cause pain, numbness, tingling, or weakness in your back, arms, or legs. The surgery “decompresses” the spine by removing or reshaping the part that’s pressing on the nerves.

**More Medical Detail**

- **Purpose:** Reduce nerve compression to improve symptoms like sciatica, back pain, or weakness.
- **Common techniques:**
  - **Laminectomy:** Removing part of the vertebral bone (lamina) to make more space.
  - **Discectomy:** Removing part of a damaged disc.
  - **Foraminotomy:** Enlarging the openings (foramina) where nerve roots exit the spine.
- **Where it’s done:** Can be in the neck (cervical), upper back (thoracic), or lower back (lumbar), depending on where the problem is.
- **Outcome:** Often reduces pain and improves mobility, though recovery time and success depend on the underlying condition and patient health.

If you like, I can also give you a **simple diagram showing where spinal decompression happens** so it’s even easier to picture. Would that help?

**Attempt 5**

Sure — let’s break it down into plain language first, then go into the more medical details.

**Plain Explanation**

“Spinal decompression surgery” is a type of operation done to relieve pressure on the spinal cord or on nerves that come out of the spine.
This pressure might be caused by things like:

- A bulging or herniated disc (the “cushions” between your back bones)
- Bone spurs (extra bits of bone that grow with age or arthritis)
- Thickened ligaments
  When nerves or the spinal cord are squeezed, it can cause pain, numbness, tingling, or weakness — often in your back, legs, or arms. The surgery aims to free up the space so nerves can work normally again.

**Medical Details**

- **Goal**: Remove or reduce whatever is pressing on the neural structures (spinal cord/nerve roots).
- **Common techniques** include:
  - **Laminectomy** – removing part of the bone (lamina) at the back of the vertebra to create more space.
  - **Discectomy** – removing part of a damaged disc that’s pushing on a nerve.
  - **Foraminotomy** – enlarging the opening where a nerve exits the spinal canal.
- **Conditions treated**: Spinal stenosis, herniated discs, degenerative disc disease, some spinal injuries or tumors.
- **Possible benefits**: Pain relief, improved movement, reduced numbness or weakness.
- **Risks** (like any surgery): Infection, bleeding, nerve injury, incomplete symptom relief.

If you like, I can also **illustrate the difference** between a compressed nerve and one after decompression surgery — that makes it much easier to understand visually. Would you like me to create that diagram?
