# Supplementary material for: ChatGPT-5.4 in health education: inter-generation stability and persistent readability challenges
Source: Front Public Health. 2026 Jul 1;14:1864894. doi: 10.3389/fpubh.2026.1864894 (PMC13369432; doi:10.3389/fpubh.2026.1864894)
Supplement: Supplementary file 2 [file Table_2.DOCX]

Additional 2 Readability and Quality results

**Table S1 Comparative Analysis of the Five Response for Q1 (Lumbar Disc Herniation Surgery)**

| **Dimension** | **Response 1** | **Response 2** | **Response 3** | **Response 4** | **Response 5** |
| --- | --- | --- | --- | --- | --- |
| **Language Level** | Starts simple, then detailed | Starts simple, then more technical | Starts simple, then detailed | Starts simple, then detailed | Entirely popular-science style |
| **Information Depth** | Brief description of indications; 3 surgical types (Discectomy/Microdiscectomy/Laminectomy); simple recovery info (days → months) | Includes urgent cases (cauda equina syndrome); 3 types + Spinal Fusion; no specific recovery time | Emphasizes functional impairment; 3 types (plus Endoscopic); recovery timeframe described | Gives specific timeline (6–12 weeks ineffective conservative treatment); 3 types (ordered by prevalence); recovery time + success rate | General description; 3 types (no Endoscopic); brief recovery note |
| **Structural Clarity** | Clear “simple → detailed” structure; medium length | Clear “simple → detailed” structure; relatively long with high information density | Clear “simple → detailed” structure; relatively long with detailed explanations | Clear “simple → detailed” structure; long with well-segmented logic | Fully popular-science flow; medium-short length |
| **Additional Content** | Mentions diagram option | Mentions diagram option | Suggests pros/cons comparison | Mentions diagram + success rate | Mentions diagram option |

**Table S2 Comparative Analysis of the Five Response for Q2 (Spinal Fusion Surgery)**

| **Dimension** | **Response 1** | **Response 2** | **Response 3** | **Response 4** | **Response 5** |
| --- | --- | --- | --- | --- | --- |
| **Language Level** | Fully plain-language explanation with everyday analogies, aimed at non-medical readers | Starts plain, then shifts to more technical detail, including terms such as *arthrodesis*, *internal fixation* | Clear everyday-language explanation with limited anatomical and pathological terminology | Fully plain language with minimal basic terms, suited for general science communication | Plain-language explanation with vivid analogies (e.g., “like welding”), minimal technical terminology |
| **Information Depth** | Overview of definition, indications, surgical steps, and recovery period | Adds bone graft types (autograft/allograft/synthetic), fixation methods, fusion process, and risks | Adds categories of underlying conditions (degeneration, deformity, trauma, stenosis, etc.), detailed steps, and postoperative functional changes | Covers definition, indications, surgical techniques, and rehabilitation, but with moderate detail | Includes surgical approaches, preparation steps, bone graft and fixation details, plus brief pros/cons and risks |
| **Structural Clarity** | Numbered sections covering term breakdown, definition, indications, surgical method, outcomes, and recovery | Structured as “plain explanation – medical definition – indications – key points” | Numbered format: “simple explanation – indications – steps – postoperative effects” | Paragraphs divided into “definition – indications – key points,” with concise structure | Block format: “plain explanation – indications – surgical steps – pros/cons” |
| **Additional Content** | Suggests comparison with lumbar disc herniation surgery | Lists specific bone graft sources, fixation device types, and complication risks | Mentions spinal cord compression indications and provides detailed rehabilitation information | Highlights reduced flexibility and recovery time; suggests visualization | Adds alternative surgical approaches, pros |

**Table S3 Comparative Analysis of the Five Response for Q3 (Spinal Decompression Surgery)**

| **Dimension** | **Response 1** | **Response 2** | **Response 3** | **Response 4** | **Response 5** |
| --- | --- | --- | --- | --- | --- |
| **Language Level** | Plain explanation as “relieving pressure on the spinal cord or nerves,” with simple listing of causes, aimed at general readers | Starts with plain explanation, then introduces basic medical terms (e.g., *spinal cord*, *spinal canal*) | Everyday description combined with limited anatomical terms, direct tone | Starts plain, followed by concise medical terminology, suitable for general science communication | Uses analogy and everyday language (e.g., “cushions” for discs), minimal technical terminology |
| **Information Depth** | Lists common causes, goals, symptoms, four main surgical types, mentions indications and applicable spinal regions | Adds explanation of causes, common techniques, surgical goals, and urgent cases (e.g., bladder/bowel dysfunction) | More detailed, including pathophysiology, surgical techniques (with rare type *corpectomy*), surgical goals, and risk list | Covers causes, common techniques, surgical goals, and applicable regions, but omits rare techniques | Covers causes, common techniques, indications, potential benefits, and risks, with concise structure |
| **Structural Clarity** | Segmented format listing causes, surgical goals, techniques, and key points | Block format organized by cause, surgical method, goal, and indications | Numbered structure outlining “why it’s done – what surgery does – types – benefits – risks” | Dual-layer structure with plain explanation plus medical detail, logically clear | Dual-layer structure with plain explanation plus medical detail, lists benefits and risks |
| **Additional Content** | Adds surgical indications, applicable spinal regions (cervical/thoracic/lumbar), recovery factors, and comparison with other surgeries | Adds urgent indications and visualization suggestion | Adds rare technique (*corpectomy*), context for combined spinal fusion, and risk list | Notes applicability to different spinal regions and suggests visualization | Expands disease scope (degenerative, traumatic, neoplastic), suggests visualization |

**Table S4 Jaccard similarity**

| **Question** | **Mean pairwise Jaccard similarity** | **Jaccard interpretation** |
| --- | --- | --- |
| **Q1** | 63.48% | High overlap |
| **Q2** | 56.32% | Moderate overlap |
| **Q3** | 54.1% | Moderate overlap |

**Table S5 Readability result**

| **Question** | **Response( Times)** | **Metric** | **Score** |
| --- | --- | --- | --- |
| Q1 | 1 | Flesch Kincaid Reading Ease | 65.5 |
| Q1 | 1 | Flesch Kincaid Grade Level | 7.1 |
| Q1 | 1 | SMOG Index | 7.2 |
| Q1 | 1 | Sentences | 21 |
| Q1 | 1 | Words | 250 |
| Q1 | 1 | Number of complext words | 31 |
| Q1 | 1 | Percent of complex words | 12.40% |
| Q1 | 2 | Flesch Kincaid Reading Ease | 49.7 |
| Q1 | 2 | Flesch Kincaid Grade Level | 10.9 |
| Q1 | 2 | SMOG Index | 10.3 |
| Q1 | 2 | Sentences | 17 |
| Q1 | 2 | Words | 310 |
| Q1 | 2 | Number of complext words | 54 |
| Q1 | 2 | Percent of complex words | 17.42% |
| Q1 | 3 | Flesch Kincaid Reading Ease | 53.3 |
| Q1 | 3 | Flesch Kincaid Grade Level | 10.6 |
| Q1 | 3 | SMOG Index | 10.3 |
| Q1 | 3 | Sentences | 18 |
| Q1 | 3 | Words | 346 |
| Q1 | 3 | Number of complext words | 57 |
| Q1 | 3 | Percent of complex words | 16.47% |
| Q1 | 4 | Flesch Kincaid Reading Ease | 58.7 |
| Q1 | 4 | Flesch Kincaid Grade Level | 8.1 |
| Q1 | 4 | SMOG Index | 8.1 |
| Q1 | 4 | Sentences | 29 |
| Q1 | 4 | Words | 350 |
| Q1 | 4 | Number of complext words | 55 |
| Q1 | 4 | Percent of complex words | 15.71% |
| Q1 | 5 | Flesch Kincaid Reading Ease | 58.6 |
| Q1 | 5 | Flesch Kincaid Grade Level | 8.9 |
| Q1 | 5 | SMOG Index | 8.6 |
| Q1 | 5 | Sentences | 19 |
| Q1 | 5 | Words | 295 |
| Q1 | 5 | Number of complext words | 41 |
| Q1 | 5 | Percent of complex words | 13.90% |
| Q2 | 1 | Flesch Kincaid Reading Ease | 65 |
| Q2 | 1 | Flesch Kincaid Grade Level | 7.4 |
| Q2 | 1 | SMOG Index | 7.7 |
| Q2 | 1 | Sentences | 22 |
| Q2 | 1 | Words | 284 |
| Q2 | 1 | Number of complext words | 38 |
| Q2 | 1 | Percent of complex words | 13.38% |
| Q2 | 2 | Flesch Kincaid Reading Ease | 47.3 |
| Q2 | 2 | Flesch Kincaid Grade Level | 10.3 |
| Q2 | 2 | SMOG Index | 10.1 |
| Q2 | 2 | Sentences | 20 |
| Q2 | 2 | Words | 295 |
| Q2 | 2 | Number of complext words | 61 |
| Q2 | 2 | Percent of complex words | 20.68% |
| Q2 | 3 | Flesch Kincaid Reading Ease | 58.95 |
| Q2 | 3 | Flesch Kincaid Grade Level | 9.2 |
| Q2 | 3 | SMOG Index | 9.3 |
| Q2 | 3 | Sentences | 19 |
| Q2 | 3 | Words | 317 |
| Q2 | 3 | Number of complext words | 48 |
| Q2 | 3 | Percent of complex words | 15.14% |
| Q2 | 4 | Flesch Kincaid Reading Ease | 52 |
| Q2 | 4 | Flesch Kincaid Grade Level | 11.3 |
| Q2 | 4 | SMOG Index | 10.5 |
| Q2 | 4 | Sentences | 10 |
| Q2 | 4 | Words | 212 |
| Q2 | 4 | Number of complext words | 33 |
| Q2 | 4 | Percent of complex words | 15.57% |
| Q2 | 5 | Flesch Kincaid Reading Ease | 50.9 |
| Q2 | 5 | Flesch Kincaid Grade Level | 12.5 |
| Q2 | 5 | SMOG Index | 10.6 |
| Q2 | 5 | Sentences | 12 |
| Q2 | 5 | Words | 304 |
| Q2 | 5 | Number of complext words | 40 |
| Q2 | 5 | Percent of complex words | 13.16% |
| Q3 | 1 | Flesch Kincaid Reading Ease | 13.6 |
| Q3 | 1 | Flesch Kincaid Grade Level | 23.7 |
| Q3 | 1 | SMOG Index | 17.6 |
| Q3 | 1 | Sentences | 5 |
| Q3 | 1 | Words | 249 |
| Q3 | 1 | Number of complext words | 47 |
| Q3 | 1 | Percent of complex words | 18.88% |
| Q3 | 2 | Flesch Kincaid Reading Ease | 46.7 |
| Q3 | 2 | Flesch Kincaid Grade Level | 10.8 |
| Q3 | 2 | SMOG Index | 10.4 |
| Q3 | 2 | Sentences | 14 |
| Q3 | 2 | Words | 228 |
| Q3 | 2 | Number of complext words | 45 |
| Q3 | 2 | Percent of complex words | 19.74% |
| Q3 | 3 | Flesch Kincaid Reading Ease | 53.5 |
| Q3 | 3 | Flesch Kincaid Grade Level | 10.1 |
| Q3 | 3 | SMOG Index | 10.2 |
| Q3 | 3 | Sentences | 16 |
| Q3 | 3 | Words | 278 |
| Q3 | 3 | Number of complext words | 49 |
| Q3 | 3 | Percent of complex words | 17.63% |
| Q3 | 4 | Flesch Kincaid Reading Ease | 50 |
| Q3 | 4 | Flesch Kincaid Grade Level | 11.2 |
| Q3 | 4 | SMOG Index | 10.6 |
| Q3 | 4 | Sentences | 12 |
| Q3 | 4 | Words | 239 |
| Q3 | 4 | Number of complext words | 40 |
| Q3 | 4 | Percent of complex words | 16.74% |
| Q3 | 5 | Flesch Kincaid Reading Ease | 51.3 |
| Q3 | 5 | Flesch Kincaid Grade Level | 10.9 |
| Q3 | 5 | SMOG Index | 10.4 |
| Q3 | 5 | Sentences | 13 |
| Q3 | 5 | Words | 250 |
| Q3 | 5 | Number of complext words | 42 |
| Q3 | 5 | Percent of complex words | 16.80% |

**Table S6 Qualitiy results (DISCERN score)**

| **Question** | **Response (Times)** | **Reviewer 1** | **Reviewer 2** | **Mean** |
| --- | --- | --- | --- | --- |
| Q1 | 1 | 38.00 | 39.00 | 38.50 |
| Q1 | 2 | 44.00 | 41.00 | 42.50 |
| Q1 | 3 | 46.00 | 43.00 | 44.50 |
| Q1 | 4 | 48.00 | 46.00 | 47.00 |
| Q1 | 5 | 44.00 | 43.00 | 43.50 |
|  |  |  | Mean | 43.2 |
| Q2 | 1 | 49.00 | 47.00 | 48.00 |
| Q2 | 2 | 44.00 | 44.00 | 44.00 |
| Q2 | 3 | 42.00 | 42.00 | 42.00 |
| Q2 | 4 | 43.00 | 42.00 | 42.50 |
| Q2 | 5 | 46.00 | 50.00 | 48.00 |
|  |  |  | Mean | 44.9 |
| Q3 | 1 | 44.00 | 44.00 | 44.00 |
| Q3 | 2 | 42.00 | 42.00 | 42.00 |
| Q3 | 3 | 47.00 | 48.00 | 47.50 |
| Q3 | 4 | 43.00 | 42.00 | 42.50 |
| Q3 | 5 | 41.00 | 42.00 | 41.50 |
|  |  |  | Mean | 43.5 |
